# Manifestations and Lived Experiences of Structural Racism for Racial and Ethnic Minority Communities Affected by HIV Across the United States

**DOI:** 10.1177/24731242251375878

**Published:** 2025-09-10

**Authors:** Betelhem A. Muno, Mariam Haris, Amra Zegeye, Rahel Behailu, Shukri A. Hassan, Jessica Y. Islam, Martez D. Smith, Bridgette Picou, Chioma Nnaji, Jennifer Barnes-Balenciaga, Marissa Miller, Steven Sawyer, Dana McCullough, Luis Mares, Ulysses Burley III, Phyllis Bijole, Ian L. Haddock, Bill (William) Hall, Mitchell Warren, Dustin T. Duncan, Stephaun Elite Wallace-Marc Jacobs, Marlene Camacho-Rivera, Jessica Jaiswal, Rena C. Patel

**Affiliations:** ^1^ Department of Medicine, University of Alabama, Birmingham, Alabama, USA.; ^2^ UW CoLab for Community & Behavioral Health Policy, University of Washington, Seattle, Washington, USA.; ^3^ Independent Researcher, Washington, District of Columbia, USA.; ^4^ YMCA of Greater Seattle, Seattle, Washington, USA.; ^5^ School of Public Health, Columbia University, New York, New York, USA.; ^6^ Department of Cancer Epidemiology, H. Lee Moffitt Cancer Center and Research Institute, Tampa, Florida, USA.; ^7^ Department of Oncologic Sciences, University of South Florida, Tampa, Florida, USA.; ^8^ Keeping Ballroom Community Alive Network, New York, New York, USA.; ^9^ The Well Project, New York, New York, USA.; ^10^ Multicultural AIDS Coalition, Boston, Massachusetts, USA.; ^11^ Keeping Ballroom Community Alive Network, New York, New York, USA.; ^12^ Trans Solutions Research and Resources Center, Indianapolis, Indiana, USA.; ^13^ POCAAN, Seattle, Washington, USA.; ^14^ HIPS Center for Health and Achievement, Washington, District of Columbia, USA.; ^15^ Latino Commission on AIDS, New York, New York, USA.; ^16^ UBtheCure, Chicago, Illinois, USA.; ^17^ HIPS Center for Health and Achievement, Washington, District of Columbia, USA.; ^18^ Normal Anomaly, Houston, Texas, USA.; ^19^ Seattle Indian Health Board, Seattle, Washington, USA.; ^20^ AVAC, New York, New York, USA.; ^21^ Department of Epidemiology, Columbia University, New York, New York, USA.; ^22^ External Relations, Fred Hutchinson Cancer Research Center, Seattle, Washington, USA.; ^23^ Department of Community Health Sciences, SUNY Downstate Health Sciences University, Brooklyn, New York, USA.; ^24^ Department of Medicine, University of Alabama, Birmingham, Alabama, USA.

**Keywords:** structural racism, HIV, COVID-19, Black, intersectionality

## Abstract

**Objective:**

To elucidate some of the manifestations of structural racism as a root cause of racialized inequities in HIV in the context of COVID-19, centered through the lens of community members with lived experiences.

**Methods:**

We partnered with eight community-based organizations to conduct focus group discussions structured around COVID-19 and HIV-related experiences. We utilized inductive coding and thematic analysis.

**Results:**

We conducted 10 focus group discussions (98 participants) across the United States between February and May 2023; 65% were ages 18–39, over 90% identified as Black, 39% were female, and 66% were cisgender. First, participants emphasized that structural racism intersects with other systems of oppression. Second, three main themes emerged as manifestations of structural racism: (1) lack of representation in state and federal decision-making levels, (2) differential access to resources, and (3) intergenerational mistrust and trauma.

**Conclusion:**

The intersecting impact of the HIV epidemic and COVID-19 pandemic underscores the pervasive effects of structural racism that manifests in the United States.

**Health Equity Implications:**

More than ever, researchers must champion the experiences and needs of racial and ethnic minority communities to affect structural change.

## Introduction

In the United States (U.S.), race shapes social stratification, with structural racism influencing where people live, work, and make health decisions.^1^ Structural racism refers to “the totality of ways in which societies foster racial discrimination, via mutually reinforcing inequitable systems … that in turn reinforce discriminatory beliefs, values, and distribution of resources.”^2^ It limits access to housing, education, employment, and health care for underrepresented racial populations.^3^ The COVID-19 pandemic intensified racialized inequities already present in the U.S. HIV epidemic.^4^ For instance, Black (42%) and Latiné (29%) people represent a disproportionate portion of the estimated 1.2 million people living with HIV (PWH)^5^ despite comprising only 13% and 19% of the total U.S. population, respectively.^5,6^ The COVID-19 age-adjusted mortality rates exhibited a similar trend for Black (21.5%) and Latiné (27%) people.^7^ The pandemic brought renewed attention to the social determinant of health (SDoH) and their roots in structural racism.^8,9^

SDoH are “the conditions in the environment where people are born, live, learn, work, play, worship, and age that affect a wide range of health, functioning, and quality-of-life outcomes and risks.”^10^ Healthy People 2030 outlines five overarching domains: education, health care, neighborhood and built environment, economic stability, and social context.^10^ These factors shape the lived experiences of PWH and individuals vulnerable to HIV as they navigate the need for medication for the rest of their lives, manage HIV-related side effects, mental health, and stigma, and confront the potential for loss of income and isolation from friends and family.^11,12^ Compared with White non-Hispanic/Latiné (hereinafter termed “White”) PWH, Black and Hispanic/Latiné (hereinafter termed “Latiné”) PWH have lower rates of viral suppression and are more likely to be unemployed, have a disability, experience homelessness, and be incarcerated; American Indian/Alaska Native and multiracial Latiné PWH experience even higher rates of homelessness.^13,14^ Black and Latiné communities have the highest rates of new HIV diagnoses but the lowest use of preexposure prophylaxis, influenced by lack of insurance, immigration status, and socioeconomic barriers.^15,16^ The significance of the SDoH in shaping health outcomes for communities affected by HIV cannot be overemphasized. Growing scholarly and policy attention recognizes that structural racism is not merely a backdrop but a fundamental driver of the inequitable distribution of these determinants.^8,9,12,17–19^

Despite the outsized role structural racism plays in the U.S. HIV epidemic, the operationalization and conceptualization of structural racism in HIV research, especially as it relates to the processes for how structural racism manifests within the lived experiences of impacted communities, is understudied in the HIV field.^19,20^ Literature exists regarding experiences of structural racism in HIV, but it is largely limited to those manifestations at the individual and not structural level.^21–28^ Furthermore, the role of community-research partnerships and engagement in codeveloping tailored interventions to address various health inequities became more critical during COVID-19.^29^ Thus, to address the gap with the conceptualization of structural racism, especially at the structural level, through the lens of communities affected by HIV, and now in the context of the COVID-19 pandemic, where these communities experienced a “double hit,” we worked with HIV-focused community-based organizations (CBOs).

## Methods

### Overall team members and positionality

The study team—including academic researchers (from African and South Asian immigrant communities), research collaborators (one multiracial and one from the African immigrant community), study staff (from African and South Asian immigrant communities), and community partners (from Black, African immigrant, Latiné, and LGBTQIA+ communities)—met regularly during project planning, implementation, analysis, and dissemination (see Supplementary Data).

We used a multifaceted approach to engage scientific and community partners through (1) a scientific advisory committee (SAC) including members with lived experiences and expertise as community members and/or health scientists and (2) qualitative data collection, in collaboration with CBO partners, examining how structural racism and SDoH shape racial/ethnic health disparities in HIV and COVID-19. Our aim was to meaningfully engage communities in cocreating and coleading research agendas in data science, specifically as it relates to HIV-related health equity research. This article presents the first of two focus areas: structural racism-related findings from our qualitative data collection and analysis efforts.

### Sampling and guide development

Through convenience sampling, sourced from a study team member’s social and professional network (S.E.W.-M.J.), we partnered with eight CBOs to conduct focus group discussions (FGDs) with lay community members affected by HIV (Table 1). The facilitators from the CBOs had existing relationships with the FGD participants through peer-to-peer interactions, community advisory memberships, and/or other interactions within their respective organization. The CBOs recruited and conducted the FGDs in English on Zoom, with individual participants joining from various locations in the U.S. We developed the FGD guide iteratively with our scientific and community partners. While the overall focus of the FGD guide was on SDoH and HIV/COVID-19, occasional questions were asked about their relationships with structural racism.

**Table 1.** Community-Based Organization Partners

Organization name	Organization website	Organization representative	Organization reach and focus	Values and missions (*excerpts based on the organization’s respective websites*)
Helping Individual Prostitutes Survive (HIPS)	www.hips.org	Phyllis Bijole Dana McCullough	Washington, DC Communities impacted by sex work and drug use	“HIPS advances the health rights and dignity of people and communities impacted by sex work and drug use by providing non-judgmental harm reduction services, advocacy, and community engagement led by those with lived experience. We envision a world where all people can use their power to live healthy and self-determined lives free from stigma violence, criminalization or oppression.”
Keeping Ballroom Community Alive Network (KBCAN)	www.KBCAN.org	Jennifer Barnes-Balenciaga Martez Smith	National House ball community, Black LGBTQ youth and young adults	“The Keeping Ballroom Community Alive Network (KBCAN) seeks to actualize liberation through confronting systemic oppression by building power, organizing ballroom styled direct actions for narrative change, and connecting the House and Ballroom Community (HBC) to resources. KBCAN will educate and advocate through radical love by addressing trauma, emphasizing health and wellness, protecting safe and sacred space, and intentionally lifting up the self-care and self-determination of the House and Ballroom Community”
Multicultural AIDS Coalition	www.Mac-Boston.org	Chioma Nnaji^a^ Agatha Adigwe	Boston, MA African born community engagement	“Since 1989, the MAC’s community mobilization efforts have focused on ensuring high quality, accessible HIV/STI prevention, care, and treatment services. The MAC’s mission is to mobilize communities of color to end the HIV epidemic. We support broader efforts to eradicate conditions that fuel the epidemic, including substance abuse, sexually transmitted infections, lack of healthcare access, homelessness, incarceration, and discrimination based on race, sex, ethnicity, sexual orientation and gender identity. Services include: HIV/Hep C/STI testing, Health System Navigation, PrEP Access & Navigation, Pharmacy Services, Drop-In Centers, Client Advocacy & Support”
Normal Anomaly	www.normalanomaly.org	Ian L. Haddock	Houston, TX Black LGBTQ, PLWHA	“Centering Black, queer plus persons to overcome barriers, end stigma and problematic narratives to actualize a new normal. To see the forward mobility of Black, queer plus persons. The Normal Anomaly has understood the need to express our narratives honestly and innovatively while changing our stories to collective resourcing and community empowerment.”
Trans Health Solutions Center	www.transsolutionsrrc.org	Marissa Miller^a^	National Transgender health, resources and education, advocacy	“TSRRC’s Mission is to bring appropriate empowerment to TGNC disenfranchised communities through a multi-tiered approach accessing resources to Comprehensive Health and Social Services. Our Vision is to offer practical solutions through a distribution of services via online and networked resources that focus directly to address the social determinants of health, empower TGNC marginalized populations through education, equity and mentorship, and to promote safety and foster leadership opportunities.”
UBtheCure	www.ubthecure.com	Dr. Ulysses Burley	National Faith focused health promotion	“A consulting company on the intersection of Health and Human Rights. A proprietary consulting company on the intersection of Faith, Health, and Human Rights”
The Well Project	www.thewellproject.org	Bridgette Picou^a^	National with some international work Women living with HIV or vulnerable to HIV	“The Well Project is a non-profit organization whose mission is to change the course of the HIV/AIDS pandemic through a unique and comprehensive focus on women and girls. The Well Project envisions a world in which women living with or vulnerable to HIV have the information, support, and tools they need to advocate for their health and well-being, and live free from stigma. Our work centers Black women, Latinas, and other women of color living with HIV across the gender spectrum while creating spaces and opportunities for all”
People of Color Against AIDS Network (POCAAN)	www.pocaan.org	Steven Sawyer^a^ Shas Carr	Seattle, WA Promote health and educational services for people of color	“Established in 1987, POCAAN is a multicultural, multi-social service agency serving marginalized communities in Seattle and greater King County including Latino, Black, Asian/Pacific Islander and Native Americans. For many years our work has been rooted in HIV/AIDS prevention, but it has grown with the understanding that related issues such as substance abuse, incarceration, homelessness, sexually transmitted diseases, racism, sexism and homophobia also contribute to community marginalization and health disparities. POCAAN is committed to providing comprehensive, multicultural awareness and prevention programs aimed at addressing health disparities experienced in marginalized communities. Vision: A sustainable, dynamic health advocacy organization able to meet the needs of 21st century clients and community. Providing services that are culturally competent and its ability to reach diverse populations. Remaining focused on building community and always remembering the human element.”

Connections with CBOs were made by Dr. Stephan Wallace through previously established relationships in other work.

There were a total of 10 FGDs conducted across the 8 organizations we partnered with (Trans Health Solutions Center and KBCAN each held two FGDs).

^a^ Indicates individuals also included as community and/or scientific members in the SAC.

CBO, community-based organization; FGDs, focused group discussions; SAC, scientific advisory committee.

### Data collection

We conducted eligibility screening and collected informed consent and sociodemographic data from each participant at the beginning of the FGDs. FGDs were audio-recorded, and a study team member (B.A.M.) took field notes when available. The recordings were transcribed by a small Black, women-owned business, reflecting a commitment to supporting and amplifying marginalized voices within the broader research process. Study team members who attended the FGDs reviewed each de-identified transcript for overall accuracy against their recall.

### Analysis

Sociodemographic data were descriptively summarized using R Studio software (version 2022.02.3-492). Two study team members (M.H. and B.A.M.) conducted inductive coding of all transcripts using Dedoose (version 9.0). We developed a codebook to generate analysis memos, facilitating the identification of preliminary major themes. To ensure the trustworthiness and relevance of our findings, we engaged with both scientific and community partners through monthly SAC meetings and CBO feedback sessions, respectively. These discussions provided critical perspectives, allowing us to assess the congruence between our findings and the lived experiences of populations affected. While no conceptual models for structural racism guided data collection, *post hoc*, we sought a model or framework that resonated with our findings. While some models or frameworks exist for articulating the role of structural racism for specific populations, for example, gender- and immigration status-based, race and ethnicity, or for a topic (e.g., maternal health), none holistically captured the unique experiences highlighted by our findings.^30–32^ We employed a “tree” analogy to help better synthesize our findings juxtapositioning structural racism against other related constructs. In this analogy, the tree symbolizes intersectionality,^33–35^ starting with the “soil” or cultural determinants, “roots” or the structural determinants (in this case structural racism), “branches” or the social determinants of health, and ending with “fruits” or health and well-being of communities.^36^

### Ethical review

This study was approved by the University of Washington’s institutional review board. All data were anonymized to ensure confidentiality.

## Results

### Participant and organization characteristics

We conducted a total of 10 FGDs across 8 CBOs, based in U.S. cities such as Washington, D.C., Boston, Seattle, and Houston, between February and May 2023, with two of the organizations conducting two FGDs each.

Though more individuals joined the FGDs, only 98 participants completed our survey, with 6–15 participants within each FGD (Table 2). About 65% of the participants were between the ages of 18 and 39, over 90% were Black, and 66% were cisgender. Twenty-three percent of the participants had some high school education, 30% had some college degree, and 25% had a bachelor’s or master’s degree. A majority (80%) indicated that racial and ethnic disparities existed in both HIV prevention and treatment. Similarly to the CBOs, all participants reported that their affiliated organizations served individuals who belonged to underrepresented populations of racial, ethnicity, sexual orientation, and/or gender and were underserved in HIV-related prevention, testing, treatment, advocacy, and research.

**Table 2.** Demographic Information of CBO FGD Participants

Characteristics	Count *N* = 98 (100%)	Characteristics	Count *N* = 98 (100%)
Age Range		Racial/ethnic Identity	
18–29	31 (31.63)	Latinx/o or Hispanic	5 (5.10)
30–39	34 (34.69)	Black/African/African American	92 (93.87)
40–49	12 (12.24)	Asian/Asian American	3 (3.06)
50–59	7 (7.14)	Native American/Native Hawaiians or other Pacific Islander/Alaska Native	2 (2.04)
60+	4 (4.08)	White American	3 (3.06)
Missing	10 (10.2)	Other	1 (1.02)
Place of Birth		Preferred Language (s)	
North America	79 (80.6)	English	93 (94.9)
The Caribbean	3 (3.06)	English and Swahili	1 (1.02)
South America	1 (1.02)	English and Spanish	1 (1.02)
Africa	12 (12.24)	Swahili	1 (1.02)
Middle East	1 (1.02)	Igbo	1 (1.02)
Missing	2 (2.04)	Missing	1 (1.02)
Sex Assigned at Birth		Current Gender	
Male	57 (58.16)	Cisgender Man	35 (35.71)
Female	38 (38.77)	Cisgender Woman	31 (31.63)
Prefer Not to Answer	3 (3.06)	Transgender Woman	19 (19.38)
		Non-binary/Non-Conforming/Gender-queer	8 (8.16)
		Another Gender	1 (1.02)
		Prefer Not to Answer	4 (4.08)
Sexual Orientation		Educational Background	
Homosexual/gay/lesbian/same gender loving	25 (25.51)	No formal educational credential	2 (2.04)
Bisexual	13 (13.26)	High school diploma or equivalent	23 (23.46)
Queer	9 (9.18)	Some college, no degree	29 (29.59)
Heterosexual/straight	32 (32.65)	Postsecondary nondegree award/Associate’s Degree	1 (1.02)
Another option (write-in)	7 (7.14)	Bachelor's Degree	17 (17.34)
Prefer Not to Answer	3 (3.06)	Master's Degree	11 (11.22)
Missing	9 (9.18)	Doctorate's Degree	14 (14.28)
		Other	1 (1.02)
Religious Affiliation		HIV-related racial/ethnic disparities most experienced by community	
Christianity	37 (37.75)	Prevention	
Islam	3 (3.06)	Treatment	12 (12.24)
Judaism	1 (1.02)	Both	5 (5.10)
Non-Denominational	9 (9.180	Other	79 (80.61)
Missing	48 (48.97)		2 (2.04)
Occupation		HIV-related Organization Affiliation	
Artist	5 (5.10)	Prevention	71 (72.44)
Finance/Consulting	3 (3.06)	Testing	59 (60.20)
Transportation	1 (1.02)	Treatment	40 (40.81)
Coordinator	9 (9.18)	Disease surveillance/Monitoring	14 (13.26)
Management	8 (8.16)	Case Management	26 (26.53)
Customer Service	11 (11.22)	Advocacy	46 (46.93)
Education/Research/Clinical	11 (11.22)	Management	16 (16.32)
Social Work/Care Services	30 (30.61)	Research	19 (19.38)
Unemployed	5 (5.10)	Community-based organizations	50 (51.02)
Other	7 (7.14)	Direct patient care	8 (8.16)
Missing	8 (8.16)	Other	3 (3.16)

Count *N* = 98 (100%) is the count of people that filled out the form, but more individuals attended the FGDs. Groups that had 0% were removed from the table including: South and East Asia birthplace, Intersex, Another Sex, Transgender Man, Arab and other Middle Eastern American.

CBO, community-based organization; FGDs, focused group discussions.

### Overview of findings

We present our findings below (see Table 3 for illustrative quotes). Most of our FGD participants self-identified as Black; however, the themes that emerged extended across racially and ethnically minoritized communities. Thus, we have chosen to use the collective term “communities of color,” while recognizing that not all communities were represented well in our data collection. Participants first described structural racism as embedded within intersectional systems of oppression that included structural sexism, homophobia, xenophobia, and more. Three key themes emerged as manifestations of structural racism: (1) racially underrepresented communities lack power and representation in decision-making at the federal and state levels, leading to negative downstream effects on insufficient and weak structural resources; (2) the need to address differential access to resources, which prioritizes access and well-being of White communities over communities of color; and (3) perpetuation of intergenerational mistrust and trauma, particularly within the various U.S. health care systems.

**Table 3.** Themes, Subthemes, and Notable Quotes from the Focus Group Discussions from the Sub-Study “Building Community Partnerships for Big Data Science: community Engagement in N3C for HIV Research” Conducted Between February and May 2023

Theme	Subtheme	Notable quotes
**Theme 0:** Overarching context	**Subtheme 0A**. Multiple systems of oppression	“Some people, African immigrants kept going to work with COVID-19 because they didn’t want to cut off the cash flow. They wanted to keep making the money and they felt they did not have the papers to apply for unemployment.” (CBO 1) “That’s about being black and on top of that, we’re in the LGBT community, so it’s gonna be twice as high for things that we have to work for.” (CBO 2) “There’s a way that these institutions and the people that run these institutions, whether they’re acting intentionally or not, like are trying to keep black men and specifically like, dark skinned black men who they’ve decided look like thugs or look like a problem or can pose a problem out of their spaces. To, “Protect the rest of their clientele from, you know, this threat or whatever.” (CBO 1) “But you know, it was just like when HIV came out. It was just considered to be uh, a gay related illness for white men only. But look at the community that monkey pox was affecting, the LGBTQ community. So, you know it’s like, not only that we’re black and brown, we’re already facing racism but our whole particular uh, community was being affected. When HIV came out, it was just geared toward gay at first. Then that monkey pox” (CBO 1)
**Theme 1.** Minoritized communities lack power and representation in decision-making at the federal and state levels, leading to a negative downstream effect on sufficient infrastructure accessibility.	**Subtheme 1A.** There is a lack of power in political and decision-making spaces.	“Who’s making these decisions on who’s getting more money.” (CBO 3) “A lot of policy that tries to get white people separated from Black people […] make sure that Black people don’t have the same opportunities as white people.” (CBO 3) “We as black and brown people, we are not at the table enough […] we have other people talking for us…They just do what they wanna do with us and put us where they wanna put us.” (CBO 1) “Resources not being in the community and not being directed to the community.” (CBO 1) “People have made decisions that don’t involve community that’ve said, it’s okay for these schools to not have these resources and this information.” (CBO 1) “Someone else mentioned something about parents not being involved […] If your parent has to travel two hours outside their community for work, how present can they be?” (CBO 1) “So, when it came to COVID-19, when you think about uh, um, HIV and other sexually transmitted diseases, I feel like uh, black people, we—we have less and less opportunity to find what data we—that—that needs to be collected or how the desegregation we talked about.” (CBO 4)
	**Subtheme 1B.** Insufficient and inadequate built environment infrastructure disproportionately impacting communities of color.	“Especially in the Black community where it’s poverty stricken, um, the people within the communities, um, barely have food for groceries, barely have food for rent, barely have a car if that. So, a lot of times we’re so focused on surviving that we’re not focused on taking care of ourselves.” (CBO 5) “When you live in a state like Boston or something—something like that where it’s primarily white, there’s going to be a lot of opportunities for you to excel being HIV positive versus if you was living in Atlanta or Montgomery.”(CBO 3) “A lot of these upper scale neighborhoods, the people that live there are paying taxes for, like, the better resources as far as school and healthcare and things of that nature… So if you live in a neighborhood where most people live alone, your chance of getting COVID is much lower than if you live in a neighborhood where most people have, ah, like a lot of people in the same apartment.”(CBO 3) “Colored people are pushed out of neighborhoods when the neighborhoods are developing”(CBO 4) “Where people live in the hood, and the ghetto, like, that’s socially, you know, those structures were made for black people decades ago to pack us into these places.” (CBO 6) “They don’t give us the same books. They don’t give us the same buildings to learn in. They never have. They still don’t[…] We are educated as best as we can.” (CBO 7) “Lack of insurance or no insurance at all. No way to get to and from my appointments. Uh, lack of education and then homeless.” (CBO 1) “Like, they’re not approving them for medical and food stamps… If you don’t have insurance, it isn’t possible to get like medication for HIV.” (CBO 8) “The mere fact that I’m living with HIV, and my access may look different because I’m gonna need some more, uh, specialized, uh, services. I’m gonna also need some more support services to make sure I stay adherent to medication.” (CBO 1) “And if you don’t have a place where you can get, um, good healthcare, you might not have PrEP or other things, um, that would help you prevent getting HIV.” (CBO 3)
**Theme 2.** Structural racism enforces differential access to resources that prioritizes access and wellbeing of white communities over communities of color.	**Subtheme 2A.** Experiences of differential access to treatment, resources, and information.	“With COVID, there’s just a lot of stuff that is out of the control of individuals and it’s just because of the way we are set up that some groups have it much worse than others.” (CBO 3) “Oh, fuck, how are Black people and poorer people going to get the vaccine?” And that’s when the tents came. So, by the time we saw the tents, most—most of the richer, white people had already gotten what they need” (CBO 3) “When it’s time to treat us, we get—we’re like, at the bottom.” (CBO 1) “Black people and poor people have the worst outcomes not caused by individual level but rather people’s jobs, transportation, not being able to work from home.” (CBO 3) “To get the medication is always very, very hard to get the medication […] that is a huge barrier.” (CBO 3) “But when it’s a person of color, it’s almost like they’re shunning us away […] Why don’t we get access to those things? Why do we have to try to, always from our neighborhoods, get to the other side of town, just so [inaudible]. And then half of the time when we make it up there, and they don’t disclose everything over the phone so, if things that we’re missing, and we don’t have so it’s like a blown trip just trying to go and get services. And even when you get there, it’s still a hard time getting it.” (CBO 1) “So, definitely economic stability is a huge, extremely huge, factor.” (CBO 6) “I think, ah, education access, and the quality of it. So, the more you know, the better—the better choices you can make, the more information you have and an understanding of what your choices are—in spite of racism. If you have the education, and can understand what your choices are, then, you know, you can make choices regardless of what else is going on.” (CBO 6) “And I was a little skeptical because there was a lot hysteria just surrounding you know getting the vaccination and like how you can contract COVID and what happened and pretty much just like a whole bunch of deaths.” (CBO 5) “It’s the fact that in the news and social media played a huge part with that and not really educating people in the way they should have been educated about it…I feel like we don’t take COVID as seriously as we need to. Um, and that’s why you don’t see as many people getting tests or getting vaccines or…” (CBO 5) “I feel like sometimes we rely so much on society to educate us, when we could be taking the chance and reading up on things that could possibly affect our lives…” (CBO 4)
	**Subtheme 2B.** The needs and wellbeing of white people are prioritized above those of communities of color.	“And then when they finished serving the white people and the richer people, they were like, “Oh, fuck, how are Black people and poorer people going to get the vaccine?” And that’s when the tents came”(CBO 3) “They don’t give us the same books. They don’t give us the same buildings to learn in. They never have. They still don’t” (CBO 7) “So, just having the education, the access to be able to drive, the knowledge to even feel comfortable enough to do that, the money to put gas in my car, even when gas was expensive to damn near $6.00, still have that economic balance to be able to do that, I think it all plays a factor and still, um, overlaps when it comes to HIV. Me knowing that I have a car. I can get to a doctor’s appointment.” (CBO 1) “Like, history was repeating itself. So, when I’m looking at the social determinants of it, it still trickled down from if you had the money, that was one thing. […] But then as time went on, and—and COVID lessened, it seemed like all black and brown persons were left to deal with the mess.”(CBO 1)
**Theme 3.** Structural racism facilitates intergenerational trauma and mistrust.	**Subtheme 3A.** Historical and ongoing oppression negatively impacts multiple generations of communities of colo*r.*	“Oh, my mom always told me if I never had any insurance, then I can’t go to the doctor.” That’s not fully true, but like overall probably. But that’s also the generational fears and the generational curses that have us on a lock right now and have had us in a lock for so long. And we don’t know much of anything else until it’s brought to our attention…” (CBO 5) “I just think culture norms um, sorta, kinda limit people from accessing um, information about HIV and AIDS.[…] When I was going up, sex wasn’t something we talked about. Um, you got your education from the school. And that was—and I was lucky that I was here in high school, so I was privy to certain informations but for older people who come, and they don’t have access to that… If for example, a parent, a child’s parent is not educated uh, in HIV transmission, things like culture could play an impact as well. Um, because their parents didn’t teach them about sexually transmitted disease…” (CBO 4) “Because most of my friends who are of a different privilege, whether that’s class or their race […] they weren’t… fearing a lot of these things because of generational wealth that was like, given to them through privilege.” (CBO 2) “…I feel like I’ve learned so much from the community, from like information on where to go or what to look for. But also, on the other—on the other hand, um, ah, social context in community, we have a lot of stigma in our community around these things as well. Um, particularly at, um, active Black communities.” (CBO 3) “And then I think it’s just a lot of stigma and it’s lack of education and then, it’s like, you know, we’ve grown up with a lot of uh, we don’t talk about this in our black house. We don’t talk about that in a black house. […] I just commend the younger generation because they’re so bold now. […] You know, I know it’s hard with the trust of the medical system, like back then, how they did the black people with um, syphilis. And we kinda don’t wanna trust the system. So, when it was time for a treatment and trials, there wasn’t too many black and brown people part of those trials and treatments.” (CBO 2) “I think it, um, support system, um, whether it be family support, or outside support, um. Some people that are positive may not have that, um, family support just because of the stigma. Um, and so having, um, the external, um, support system is very important…” (CBO 6)
	**Subtheme 3B.** The harmful experiences accessing health-related services leads to mental and physical trauma and lack of trust in providers and health care systems.	“I’ve been noticing with her is she’s saying no to a lot, being stubborn because she don’t trust the healthcare system[…]And what mechanisms are in place to really address that?…”.You have to remember like your community, like the community that you’re serving. […]you know, they’re—they’re scared. They haven’t been to the doctors in ages. They don’t like coming to the doctor. […]they’re already coming in with just a negative connotation of the environment.” (CBO 3) “So I will say the healthcare and the education will kind of go hand in hand together for—for the community right now…”(CBO 8) “We need to call providers to their condescending tones and the consequent substandard treatment as that can help check the racism and make providers aware.” (CBO 4) “Some of how they just like, discount your concerns or the things that you’re trying to present to them” (CBO 4) “We as black and brown people, we already have so many different comorbidities like our diabetes, high blood pressure and all those different things. So, we have a lot more to gamble with or take chances on when it comes to different things, you know.[..] So, I just think along with the comorbidities that we already have, it kinda put another um, you know, strain on us. […]You know, it’s like, that’s why you know, trusting the medical system for black and brown people, it’s so much harder than when it comes to Caucasians…” (CBO 2) “That goes back to the distrust in the healthcare. [Inaudible—crosstalk] [00:50:10] It took me three times to catch COVID for me to get the shot.” (CBO 3) “Everything that everybody naming…history of like, trauma with the…medical industrial complex… And they made us make…real harsh, quick decisions with a lot of folks. […] So…the difference just from being white and black and like, the amount of fear that came during COVID.” (CBO 2) “I think part of the problem was communication definitely to the city to healthcare agencies…and just inconsistent kinda guidelines and recommendations from the federal government.” (CBO 2) “So, I would like if it could possibly—and I see it all the time, people that I speak to, some research on—on people that are living with HIV, or HIV and AIDs and what—what COVID caused them, and that they’re still dealing with it. They have to have a special assessment, and they still have to have something to help them through because they’re still going through it, and—and, you know, hopefully, there could be a solution because as—the way that I see it now is all these other things are very important, but we’re still going through—as human beings, collectively, a collective trauma.” (CBO 1) When it comes down to people, um, who may think that they’re at risk, who want to get tested, afraid to get tested. Um, or who may even, you know what I mean, may be living with HIV, you know what I mean. Having to deal with the stigma and the guilt and the shame and other stuff…”You need services after that. Like you need services after that. Like how am I supposed to literally, you know, get a positive test and then just go back to the ball like I just didn’t—like that just didn’t happen.” (CBO 3) Uh, and then you know, we’re already lagging. And then COVID just exacerbated the whole issues and now we’re even—it’s even worse, in our community. And so, I think we should be including mental health, as a—just as much of a priority in programming, you know when we’re talking about uh, access to HIV care, COVID care, you know, and all—all of the other things that we’re providing care around. So, mental health should be included and given a—yeah. Given priority.” (CBO 4) “A lot of the things that people associate with homelessness is—uh, mental health contributes to that. You know, so, they’re thinking, “Oh, you homeless, you dirty, you know”—no. It’s I have mental health issues—and nobody is addressing them so –no, I’m not showering. Yes, I’m using drugs. Yes, I’m out here laid up. Yes, you can say I’m—dirty because I’m not even mentally here.” (CBO 7) “I think that, it kinda go hand in hand some—with some people because if you are not stable, say uh, the lack of education, if you’re homeless, if you are not mentally capable, your mental illness, you’re on drugs. And so, if you’re dealing with all those things and there’s COVID and HIV and if you’re not taking care of one, you’re definitely gonna be able to take care of the other one. Because if I’m not having a—a stable place to take my medicine, or have a stable doctor or the insurance. I don’t have the transportation, I’m getting high, I’m drinking, I’m prostituting, I’m a street worker. All those things right now to me are very—they’re—they’re not important. They’re not important at—at this time, unless I get into care where someone takes the time to educate me. Because I’m not able to advocate and educate myself at the moment, because of my mental is not stable enough. So, it kinda combines, yeah.”(CBO 2)
	**Subtheme 3C.** Lack of representation, language barriers, accessible services, and cultural humility, sensitivity, and competency at multiple levels of the health care system.	“Making sure that there are doctors that are representative of community. Doctors not being able to relate to their clients, not knowing how to talk to trans or queer clients” (CBO 3) “Cultural competency -first interaction can make or break the outcome. Forms aren’t updated to include legal name and chosen name.” (CBO 3) “Language access and communication with providers, language can impact the care” (CBO 3) “Making sure that we have, um, doctors that are—or providers that identify with the individuals in our community or can best—hormones, and, ah, shots, things like that. […]be open to a doctor who can understand where they’re coming from, especially during like big sexual things that happened with gay men, or men who have sex with men.” (CBO 3) “That first interaction can make or break whether or not you get to someplace that you wanna go for care.” (CBO 3) “So, thinking of, um, people who don’t speak English, being able to have access to, um, to quality care available, um, in Spanish or any other language, that people might, um, speak about… So, being able to—to have quality based on—on your own, um, identity and needs. I’m also thinking about, um, training of—of staff in the clinic.[..]from the front desk person, um, to the pharmacist,[…]how they are trained to have cultural humility when it comes to working, um, with people living with HIV, um, and again, all of our various identities.” (CBO 1)

### Overarching context: Multiple systems of oppression

**FIG. 1.**
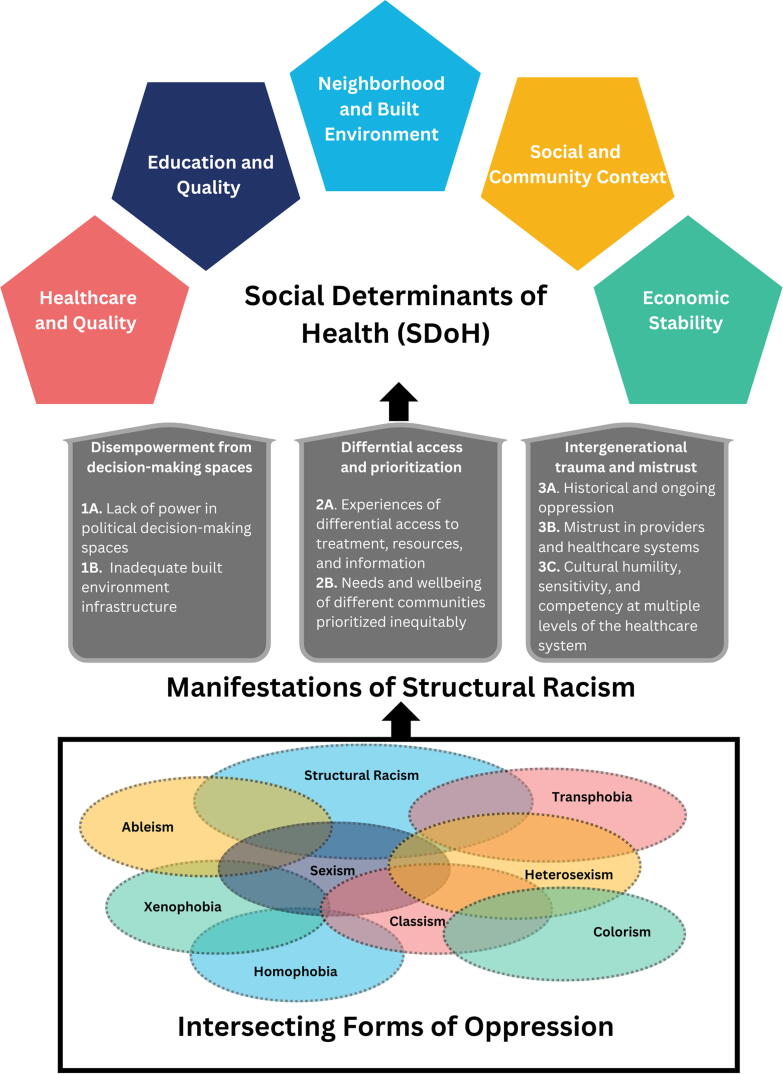
The downstream relationship between structural racism and SDoH seen through the experiences of communities living with and impacted by HIV. SDoH, social determinant of health.

Majority of participants stressed the intersectionality of each of their various social positions (e.g., being a Black immigrant woman who is gay) and the ways that the multiple systems of oppression had a downstream impact on their lives. The understanding that structural racism is intertwined with other structural forms of oppression (*intersectional* forms of oppression or the soil of the tree framework mentioned above),^36^ is fundamental to explaining how purposely harmful and insufficient infrastructure creates negative lived experiences for racially and ethnically minoritzed communities. Many highlighted the challenge of navigating the U.S. systems by describing the barriers they encountered as people who experience stigma and discrimination based on their various social positions. Others spoke of the ways they experienced intersectional discrimination and racism—such as being Black and queer, Black and trans, or young and dark-skinned—shaping the ways they interacted with health care institutions and health information at the individual level. Many highlighted the connection between their individual experiences with HIV and COVID-19 sharing the paralleling ways structural racism affected them and their communities (e.g., misinformation and disinformation, differential resource levels, limited health care system capacity), which caused a unique experience due to already having increased health vulnerability, the historical trauma of living with HIV, and experiencing multiple forms of stigma due to being affected by HIV. Figure 1 captures the relationship between structural racism and SDoH through the manifestations highlighted by the participants paralleling the structure of the tree framework. Next, we discuss the three main themes of structural racism manifestations while showing the subthemes with illustrative quotes (and detailed analysis in Supplementary Data).

#### Theme 1: Communities of color lack power and representation in decision-making at the federal and state levels, leading to a negative downstream effect on sufficient infrastructure

Participants expressed a sense of disempowerment in decision-making spaces, noting that those in power do not represent their communities or prioritize their needs (subtheme 1A). One participant said, *“Who’s making these decisions on who’s getting more money”* (CBO 3). The impact of health inequities, laid painfully bare during the COVID-19 pandemic, showed the disparity in resources and opportunities between communities. They frequently shared how they have observed that differential power then translates to financial differences between White and affluent communities receiving better resources and information. In turn, this translates to inadequate infrastructure and intentional systemic neglect of their neighborhoods (subtheme 1B), as one participant pointed out, “*Colored people are pushed out of neighborhoods when the neighborhoods are developing”* (CBO 4).

#### Theme 2: Structural racism enforces differential access to resources that prioritizes access and well-being of White communities over communities of color

Participants largely expressed disappointment, anger, and disdain when discussing their experiences of unequal access to resources during the COVID-19 pandemic (subtheme 2A). A participant described how *“When it’s time to treat us,… we’re like, at the bottom.”* (CBO 1). These experiences caused feelings reminiscent of personal and historical experiences of HIV but just more evident with COVID-19. Many participants’ narratives emphasized the role of white supremacy and overt structural racism in determining which communities were prioritized (subtheme 2B). Another participant described, “*They don’t give us the same books. They don’t give us the same buildings to learn in. They never have. They still don’t”* (CBO 7).

#### Theme 3: Structural racism facilitates intergenerational trauma and mistrust

In addition to sharing their experiences around unequal access to power and resources, participants also described how persistent inequities have led to intergenerational trauma and mistrust (subtheme 3A). A participant shared, “*…I think it’s just a lot of stigma and it’s lack of education …We don’t talk about that in a black house. […] I just commend the younger generation because they’re so bold now…I know it’s hard with the trust of the medical system, like back then, how they did the black people with um, syphilis. And we kinda don’t wanna trust the system. So, when it was time for a treatment and trials, there wasn’t too many black and brown people part of those trials and treatments.”* (CBO 2). Importantly, participants’ examples illustrate how both individual and collective trauma have intergenerational consequences within health care (subtheme 3B). A participant described, *“That goes back to the distrust in the healthcare…It took me three times to catch COVID for me to get the shot”* (CBO 3). While increased cultural sensitivity and humility in health care settings can help address this distrust, more work at higher levels of systems must occur to truly ameliorate the mistrust (subtheme 3C). A participant suggested, “*Making sure that there are doctors that are representative of community. Doctors not being able to relate to their clients, not knowing how to talk to trans or queer clients”* (CBO 3).

## Discussion

The HIV epidemic and COVID-19 pandemic in the U.S. illustrate how structural forces perpetuate health inequities.^19,37^ Participants’ narratives revealed how structural racism manifests in people’s everyday lives, highlighting the intersectional nature of oppression, ranging from sexism, xenophobia, and ableism. Our participants articulated specific manifestations of structural racism, made more evident by COVID-19 but present with HIV too,^38^ on (1) political disempowerment, (2) unequal access to resources, and (3) intergenerational trauma and mistrust. These findings underscore the need for structural interventions to address these inequities.

While the concept of intersectional stigma is familiar in the HIV field,^39–41^ what our participants suggested was a more profound level of intersectionality existing at the structural level. This challenges us to go beyond recognizing individual-level racism (e.g., derogatory language or implicit bias)^42–44^ or historical events (e.g., slavery, Jim Crow)^1^ and appreciate that structural racism closely intersects with other forms of oppression such as sexism, xenophobia, and more.^45^ Participants articulated that the daily struggles they faced in trying to access sufficient resources were due to intersectional forms of oppression. For many immigrant and refugee people, for instance, struggles such as lack of interpreter services or fear of being undocumented were exacerbated by xenophobia.^32^

Participants easily linked structural racism to political disempowerment. They expressed frustration about lacking decision-making power at various levels, which translated to insufficient infrastructure and resources. This disempowerment directly translated to their inability to adequately care for themselves and their communities. This finding reflects known disparities in HIV and COVID-19 that are driven by structural racism.^46^ For instance, higher levels of measured structural racism were associated with increased rates of COVID-19 cases and deaths, even after accounting for factors such as sociodemographic characteristics, health care access, population density, or health.^45^

Our study describes how these statistical realities play out daily. In particular, participants articulated the emotionally and physically damaging effects of experiencing the prioritization of White communities’ health and well-being over their own communities. For instance, participants shared their experiences of observing certain White communities receive better COVID-19 testing and vaccine access, including an influx of White people to their neighborhoods when vaccination tents arrived. Within a social and economic system already structured by white supremacy, these observations demand that relevant stakeholders work harder to build structural-level interventions that address this harm.

Lastly, participants clearly linked past and ongoing trauma with mistrust—highlighting the historical context to their present-day lived experiences. Medical mistrust is broadly documented in the extant public health literature, particularly within racial and ethnic minority communities.^47^ Mistrust has been identified as barriers to engagement in HIV care and ART adherence.^48^ HIV independently adds challenges to one’s daily life through the frequent interactions with the health care system for the medical support needed, experiencing stigma, isolation, mental health challenges, and financial hardships, along with the historical and ongoing trauma still being experienced by vulnerable communities.^11,12,49^ During the height of the COVID-19 pandemic, “vaccine hesitancy” among Black individuals was often cited as a barrier to curbing the pandemic.^50^ This “hesitancy” has been mostly linked to the Tuskegee study, missing the opportunity to ground this mistrust in ongoing, daily lived experiences of social and economic exclusion for racial and ethnic minority communities.^51,52^ Our empirical findings align with previous analyses linking white supremacy and structural racism to mistrust,^52^ and specify how these factors perpetuate intergenerational harm.

### Health equity implications

Our findings support the call for structural change to address the cascade of health inequities caused by social, economic, and political oppression. We highlight some “what’s” but also some “how’s” of how to proceed with seeking structural change. Regarding the “what’s,” we stress a few suggestions within the realm of health care systems and for researchers. Health care systems need to move beyond acknowledging implicit bias; implicit bias training alone is insufficient because the onus is on individuals to effect structural change when the level of change is fundamentally beyond the individual.^53^ Carter and colleagues recommend combining efforts at the institutional and structural levels alongside individual-level training.^53^ Relatedly, cultural competency training falls short of reaching the needed structural changes; health care training must prioritize teaching cultural humility and person-centered care and engender broader inclusion and representation.^54^ Researchers attempting to address health inequities need to move beyond describing disparities and even beyond the SDoH to focus on the political and structural determinants of health at county, state, and federal levels. Fundamentally, the changes needed are more dramatic, as Alang and Blackstock have outlined in the context of COVID-19, but with broader implications: (1) redistribute resources, (2) enforce mandates that redistribute power, (3) enact legislation that guarantees support for people, (4) center experiences and voices of the most impacted communities in policy development, and (5) evaluate multidimensional effects of policies across systems. As for the “how’s,” we stress community engagement and collective action for the ultimate goal of health justice. Researchers, us included, need to move beyond community consultancy models of work to collaborations and then true partnerships.^55^ Admittedly, while hard work, collective action can only be achieved through bidirectional respect and long-standing relationships.^56^ Lastly, positive structural change is possible. Many states around the U.S. have passed, or are in the process of trying to pass legislation that criminalizes efforts to address structural racism and other diversity, equity, and inclusion topics.^57^ These policy moves have exacerbated and will continue to reinforce the inequities we aim to address, particularly through existing policies that criminalize HIV, transgender identities, substance use, and poverty.^58–60^ More than ever, now is the time to double down on this hard work.

### Limitations

While this project had many strengths, such as sampling throughout the U.S., the CBOs leading the FGD conduct, and participating in interpretation and dissemination, including as coauthors,^61^ there are a few important limitations to note. First, we did not capture the geographical distribution of the participants, and a majority of our participants were Black; we believe this may limit transferability of the findings across geography and other racial and ethnic minority communities, such as Latiné, Native American, or Asian American communities. Nonetheless, many participants felt their perspectives applied more broadly, as did our coauthor team, which did constitute members belonging to these other communities. Second, conducting these conversations via Zoom may have limited the depth of discussion or introduced accessibility challenges for some participants. However, prior research by members of this team demonstrates the feasibility of using Zoom for FGDs among older African immigrants in the U.S., even those with limited prior experience with virtual video conferencing technologies. Given this, we remain hopeful the virtual discussions were still accessible to many. Third, the participants who joined our FGDs largely had high levels of formal education and articulated themes at high levels of conceptualization and abstraction that even some research team members were not prepared to consider—case in point, how the participants raised elements related to structural racism when our FGD guides were mainly asking about the role of SDoH on HIV and COVID-19 experiences. Thus, themes articulated in this article may not resonate as readily with other lay community members.

## Conclusion

Structural racism, inextricably linked with other structural determinants, shapes the lived experiences of racial and ethnic minority communities through political disempowerment, inequitable access to resources, and intergenerational trauma. Our study provides support for the argument that the HIV and COVID-19 epidemics will not be adequately addressed without urgent action on the various ways in which structural racism manifests in the U.S. Given the troubling trend toward stifling discussions on race and racism, now is the time to accelerate, not subdue, national, state, and local discourse on structural racism and intersectional oppression. Now is not the time for silence—it is time for bold, transformative actions.

## Data Availability

Working files may be made available to individual researchers upon request.

## Notes

**Cite this article as:** Muno BA, Haris M, Zegeye A, Behailu R, Hassan SA, Islam JY, Smith MD, Picou B, Nnaji C, Barnes-Balenciaga J, Miller M, Sawyer S, McCullough D, Mares L, Burley III U, Bijole P, Haddock IL, Hall B (William), Warren M, Duncan DT, Jacobs SE W-M, Camacho-Rivera M, Jaiswal J, Patel RC (2025) Manifestations and lived experiences of structural racism for racial and ethnic minority communities affected by HIV across the United States, *Health Equity* 9:1, 474–490, DOI: 10.1177/heq.2025.0039.

## Abbreviations Used

ART: Antiretroviral therapy
CBOs: Community-based organizations
FGD: Focus group discussions
HIV: Human immunodeficiency virus
PWH: People living with HIV
SAC: Scientific advisory committee
SDoH: Social determinants of health
U.S.: United States

## References

[1] Gee GC, Hicken MT. Structural racism: the rules and relations of inequity. Ethn Dis 2021;31(Suppl 1):293–300; doi: 10.18865/ed.31.S1.293 34045831 PMC8143846

[2] Krieger N. Discrimination and health inequities. Int J Health Serv 2014;44(4):643–710; doi: 10.2190/HS.44.4.b 25626224

[3] Andrasik MP, Maunakea AK, Oseso L, et al. Awakening: the unveiling of historically unaddressed social inequities during the COVID-19 pandemic in the United States. Infect Dis Clin North Am 2022;36(2):295–308; doi: 10.1016/j.idc.2022.01.009 35636901 PMC8806123

[4] Millett GA. New pathogen, same disparities: why COVID‐19 and HIV remain prevalent in U.S. communities of colour and implications for ending the HIV epidemic. J Int AIDS Soc 2020;23(11):e25639; doi: 10.1002/jia2.25639 33222424 PMC7645849

[5] Anonymous. Volume 32 | HIV Surveillance | Reports | Resource Library | HIV/AIDS. 2021| CDC. Available from: https://www.cdc.gov/hiv/library/reports/hiv-surveillance/vol-32/index.html [December 6, 2023].

[6] Anonymous. U.S. Census Bureau QuickFacts: United States. n.d. Available from: https://www.census.gov/quickfacts/fact/table/US/PST045222 [Last accessed: December 6, 2023].

[7] Anonymous. COVID-19 Provisional Counts - Health Disparities. 2023. Available from: https://www.cdc.gov/nchs/nvss/vsrr/covid19/health_disparities.htm [Last accessed: September 19, 2024].

[8] Rastetter M. MD. How Racism Is a Structural and Social Determinant of Health. 2021. Available from: https://wexnermedical.osu.edu/our-stories/racism-is-a-social-determinant-of-health [Last accessed: July 6, 2025].

[9] Tribe M. Structural Racism: The Root Cause of the Social Determinants of Health - Petrie-Flom Center. 2020. Available from: https://petrieflom.law.harvard.edu/2020/09/22/structural-racism-social-determinant-of-health/ [Last accessed: July 6, 2025].

[10] Anonymous. Healthy People 2030 | Odphp.Health.Gov. n.d. Available from: https://odphp.health.gov/healthypeople [Last accessed: January 27, 2025].

[11] Anonymous. Living with HIV. n.d. Available from: https://viivhealthcare.com/about-hiv/living-with-hiv/ [Last accessed: July 6, 2025].

[12] Yermukhanova L, Kuzembayev M, Salkhanova A, et al. Exploring socio-economic dimensions in HIV research: a comprehensive bibliometric analysis (1992–2024). Glob Health Action 2025;18(1):2474787; doi: 10.1080/16549716.2025.2474787 40071324 PMC11905308

[13] Padilla M, Luna-Gierke RE, Carree T, et al. racial differences in social determinants of health and outcomes among hispanic/latino persons with HIV—United States, 2015–2020. J Racial Ethn Health Disparities 2024;11(1):574–588; doi: 10.1007/s40615-023-01542-4 36826779 PMC10447624

[14] Adams JW, Lurie MN, King MRF, et al. Potential drivers of HIV acquisition in African-American women related to mass incarceration: an agent-based modelling study. BMC Public Health 2018;18(1):1387; doi: 10.1186/s12889-018-6304-x 30563496 PMC6299641

[15] Anonymous. AIDSVu Releases New Data Showing Significant Inequities in PrEP Use Among Black and Hispanic Americans. 2022. Available from: https://aidsvu.org/prep-use-race-ethnicity-launch-22/ [Last accessed: 9/20/2024].

[16] Campbell JT, Adams OR, Bennett-Brown M, et al. PrEP familiarity, interest, and usage among 364 black and Hispanic adults in Indiana. Front Public Health 2022;10:810042; doi: 10.3389/fpubh.2022.810042 35602152 PMC9120626

[17] Beltran RM, Holloway IW, Hong C, et al. Social determinants of disease: HIV and COVID-19 experiences. Curr HIV/AIDS Rep 2022;19(1):101–112; doi: 10.1007/s11904-021-00595-6 35107810 PMC8808274

[18] Bailey ZD, Feldman JM, Bassett MT. How structural racism works—Racist policies as a root cause of U.S. racial health inequities. N Engl J Med 2021;384(8):768–773; doi: 10.1056/NEJMms2025396 33326717 PMC11393777

[19] Bowleg L, Malekzadeh AN, Mbaba M, et al. Ending the HIV epidemic for all, not just some: structural racism as a fundamental but overlooked social-structural determinant of the U.S. HIV epidemic. Curr Opin HIV AIDS 2022;17(2):40–45; doi: 10.1097/COH.0000000000000724 35102051 PMC9109814

[20] Muno BA, Islam JY, Schwartz R, et al. Structural racism conceptualization and operationalization for research for the U.S. HIV epidemic: findings from a scoping review and implications for advancing research for structural interventions. AIDS Behav 2024;28(Suppl 1):149–165; doi: 10.1007/s10461-024-04417-9 39093355 PMC11927403

[21] Lett E, Asabor EN, Tran N, et al. Sexual behaviors associated with HIV transmission among transgender and gender diverse young adults: the intersectional role of racism and transphobia. AIDS Behav 2022;26(11):3713–3725; doi: 10.1007/s10461-022-03701-w 35661016 PMC13277673

[22] English D, Carter JA, Boone CA, et al. Intersecting structural oppression and black sexual minority men’s health. Am J Prev Med 2021;60(6):781–791; doi: 10.1016/j.amepre.2020.12.022 33840546 PMC8274250

[23] Dale SK, Dean T, Sharma R, et al. Microaggressions and discrimination relate to barriers to care among black women living with HIV. AIDS Patient Care STDS 2019;33(4):175–183; doi: 10.1089/apc.2018.0258 30932695 PMC6459277

[24] Scott HM, Pollack L, Rebchook GM, et al. Peer social support is associated with recent HIV testing among young black men who have sex with men. AIDS Behav 2014;18(5):913–920; doi: 10.1007/s10461-013-0608-8 24065436 PMC3965658

[25] Sullivan PS, Knox J, Jones J, et al. Understanding disparities in viral suppression among Black MSM living with HIV in Atlanta Georgia. J Int AIDS Soc 2021;24(4):e25689; doi: 10.1002/jia2.25689 33821554 PMC8022103

[26] Cené CW, Akers AY, Lloyd SW, et al. Understanding social capital and HIV risk in rural African American communities. J Gen Intern Med 2011;26(7):737–744; doi: 10.1007/s11606-011-1646-4 21311999 PMC3138603

[27] Small LA, Godoy SM, Lau C. Perceptions of healthcare accessibility and medical mistrust among Black women living with HIV in the USA. Cult Health Sex 2023;25(10):1295–1309; doi: 10.1080/13691058.2022.2155706 36571392 PMC10558086

[28] Freeman R, Gwadz MV, Silverman E, et al. Critical race theory as a tool for understanding poor engagement along the HIV care continuum among African American/Black and Hispanic persons living with HIV in the United States: a qualitative exploration. Int J Equity Health 2017;16(1):54; doi: 10.1186/s12939-017-0549-3 28340589 PMC5364619

[29] Stadnick NA, Cain KL, Watson P, et al. Engaging underserved communities in COVID-19 health equity implementation research: an analysis of community engagement resource needs and costs. Front Health Serv 2022;2:850427; doi: 10.3389/frhs.2022.850427 36258685 PMC9574473

[30] Volpe VV, Hoggard LS, Willis HA, et al. Anti-Black structural racism goes online: a conceptual model for racial health disparities research. Ethn Dis n.d;31(Suppl 1):311–318; doi: 10.18865/ed.31.S1.311 34045833 PMC8143849

[31] Headen I. Structural racism, geographies of opportunity, and maternal health inequities: a dynamic conceptual framework. J Racial Ethn Health Disparities 2025; doi: 10.1007/s40615-025-02345-5 PMC12631708 40029480

[32] Samari G, Wurtz HM, Abularrage TF, et al. Structural gendered racism as conceptualized by immigrant women in the United States. Soc Sci Med 2024;351(Suppl 1):116396; doi: 10.1016/j.socscimed.2023.116396 38825373 PMC11149896

[33] Crenshaw K. Demarginalizing the intersection of race and sex: a black feminist critique of antidiscrimination doctrine, feminist theory and antiracist politics. Univ Chic Leg Forum 2015;1989(1).

[34] Cho S, Crenshaw KW, McCall L. Toward a field of intersectionality studies: theory, applications, and praxis. Signs J Women Cult Soc 2013;38(4):785–810; doi: 10.1086/669608

[35] Anonymous. The Urgency of Intersectionality | Kimberlé Crenshaw | TED; 2016.

[36] Anonymous. Intersectionality: Amplifying Impacts on Health Equity. n.d. Available from: https://www.mathematica.org/blogs/intersectionality-amplifying-impacts-on-health-equity [Last accessed: September 19, 2024].

[37] National Academies of Sciences E, Medicine NA of, Nursing 2020–2030 C on the F of, et al. Social Determinants of Health and Health Equity. In: The Future of Nursing 2020-2030: Charting a Path to Achieve Health Equity National Academies Press (US). National Academies of Sciences; 2021. 34524769

[38] Bowleg L. We’re not all in this together: on COVID-19, intersectionality, and structural inequality. Am J Public Health 2020;110(7):917; doi: 10.2105/AJPH.2020.305766 32463703 PMC7287552

[39] Logie CH, James L, Tharao W, et al. HIV, gender, race, sexual orientation, and sex work: a qualitative study of intersectional stigma experienced by HIV-positive women in Ontario, Canada. PLoS Med 2011;8(11):e1001124; doi: 10.1371/journal.pmed.1001124 22131907 PMC3222645

[40] Logie C, James L, Tharao W, et al. Associations between HIV-related stigma, racial discrimination, gender discrimination, and depression among HIV-positive African, Caribbean, and Black women in Ontario, Canada. AIDS Patient Care STDS 2013;27(2):114–122; doi: 10.1089/apc.2012.0296 23373665

[41] Maragh-Bass AC, Williams T, Agarwal H, et al. Exploring stigma, resilience, and alternative HIV preventive service delivery among young men who have sex with men of color. Clin Nurs Res 2023;32(7):1046–1056; doi: 10.1177/10547738231184295 37401801 PMC11500069

[42] Díaz RM, Ayala G, Bein E. Sexual risk as an outcome of social oppression: data from a probability sample of Latino gay men in three U.S. cities. Cultur Divers Ethnic Minor Psychol 2004;10(3):255–267; doi: 10.1037/1099-9809.10.3.255 15311978

[43] Bogart LM, Landrine H, Galvan FH, et al. Perceived discrimination and physical health among HIV-Positive black and Latino men who have sex with men. AIDS Behav 2013;17(4):1431–1441; doi: 10.1007/s10461-012-0397-5 23297084 PMC3631464

[44] Lewis JA, Neville HA. Construction and initial validation of the gendered racial microaggressions scale for black women. J Couns Psychol 2015;62(2):289–302; doi: 10.1037/cou0000062 25867696

[45] Tan SB, deSouza P, Raifman M. Structural racism and COVID-19 in the USA: a county-level empirical analysis. J Racial Ethn Health Disparities 2022;9(1):236–246; doi: 10.1007/s40615-020-00948-8 33469868 PMC7815192

[46] Anonymous. Structural Racism: the Rules and Relations of Inequity - PMC. n.d. Available from: https://www-ncbi-nlm-nih-gov.offcampus.lib.washington.edu/pmc/articles/PMC8143846/ [Last accessed: September 19, 2024].

[47] Benkert R, Cuevas A, Thompson HS, et al. Ubiquitous yet unclear: a systematic review of medical mistrust. Behav Med 2019;45(2):86–101; doi: 10.1080/08964289.2019.1588220 31343961 PMC6855383

[48] El-Krab R, Brousseau N, Kalichman SC. Medical mistrust as a barrier to HIV prevention and care. J Behav Med 2023;46(6):897–911; doi: 10.1007/s10865-023-00417-7 37698802

[49] Bogart LM, Ojikutu BO, Tyagi K, et al. COVID-19 related medical mistrust, health impacts, and potential vaccine hesitancy among black Americans living with HIV. J Acquir Immune Defic Syndr 2021;86(2):200–207; doi: 10.1097/QAI.0000000000002570 33196555 PMC7808278

[50] Yuko E. Why Are Black Communities Being Singled Out as Vaccine Hesitant? 2021. Available from: https://www.rollingstone.com/culture/culture-features/covid-19-vaccine-hesitant-black-communities-singled-out-1137750/ [Last accessed: September 19, 2024].

[51] Jaiswal J, Halkitis PN. Towards a more inclusive and dynamic understanding of medical mistrust informed by science. Behav Med 2019;45(2):79–85; doi: 10.1080/08964289.2019.1619511 31343962 PMC7808310

[52] Jaiswal J, LoSchiavo C, Perlman DC. Disinformation, misinformation and inequality-driven mistrust in the time of COVID-19: lessons unlearned from AIDS denialism. AIDS Behav 2020;24(10):2776–2780; doi: 10.1007/s10461-020-02925-y 32440972 PMC7241063

[53] Anonymous. Moving Beyond Implicit Bias Training: Policy Insights for Increasing Organizational Diversity - Ivuoma N. Onyeador, Sa-Kiera T. J. Hudson, Neil A. Lewis, 2021. n.d. Available from: https://journals-sagepub-com.offcampus.lib.washington.edu/doi/full/10.1177/2372732220983840 [Last accessed: September 19, 2024].

[54] Solchanyk D, Ekeh O, Saffran L, et al. Integrating cultural humility into the medical education curriculum: strategies for educators. Teach Learn Med 2021;33(5):554–560; doi: 10.1080/10401334.2021.1877711 33573412

[55] Key KD, Furr-Holden D, Lewis EY, et al. The continuum of community engagement in research: a roadmap for understanding and assessing progress. Prog Community Health Partnersh 2019;13(4):427–434. 31866597 10.1353/cpr.2019.0064

[56] Alang S, Blackstock O. Health justice: a framework for mitigating the impacts of HIV and COVID-19 on disproportionately affected communities. Am J Public Health 2023;113(2):194–201; doi: 10.2105/AJPH.2022.307139 36521095 PMC9850620

[57] Anonymous. New Anti-DEI legislation goes into effect in 4 States. n.d. Available from: https://www.chronicle.com/article/new-anti-dei-legislation-goes-into-effect-in-4-states [Last accessed: September 23, 2024].

[58] Anonymous. The Carceral Production of Transgender Poverty: How Racialized Gender Policing Deprives Transgender Women of Housing and Safety - Dilara Yarbrough, 2023. n.d. Available from: https://journals-sagepub-com.offcampus.lib.washington.edu/doi/full/10.1177/14624745211017818 [Last accessed: September 23, 2024].

[59] Scher BD, Neufeld SD, Butler A, et al. “Criminalization causes the stigma”: perspectives from people who use drugs. Contemp Drug Probl 2023;50(3):402–425; doi: 10.1177/00914509231179226

[60] Murray TA, Oerther S, Simmons KJ. Anti-DEI legislation targeting colleges and universities: its potential impacts on nursing education and the pursuit of health equity. Nurs Outlook 2023;71(4):101994; doi: 10.1016/j.outlook.2023.101994 37336170

[61] Duncan D. Composing authorship teams for health equity: an introduction to the health equity research production model. Intr J Equity and Health n.d. 10.1186/s12939-025-02524-0 PMC12164112 40506705

