# Supplementary material for: Manifestations and Lived Experiences of Structural Racism for Racial and Ethnic Minority Communities Affected by HIV Across the United States
Source: Health Equity. 2025 Sep 10;9(1):474–90. doi: 10.1177/24731242251375878 (PMC12951217; doi:10.1177/24731242251375878)
Supplement: Supplementary Data [file 24731242251375878_supp_data.docx]

**Supplementary Materials**

**Lay Summary**

While we know that racism in systems affects the spread of HIV in the U.S. (and was also seen with COVID-19), we don't know much about how this racism in systems shows up in the lives of people who are affected. So, we did a study to talk about how people from different races in the U.S. experience racism and how it affects HIV, especially since COVID-19.

We talked to 98 people in 10 group discussions from February to May 2023. Most of them were Black, and many were between 18-39 years old. We worked with community organizations that help people from different races, sexual orientations, genders, and those who have or are at risk for HIV to recruit these people.

Three main ideas came up about how racism in systems affects people:

Theme 0. Big Picture

Racism in systems is part of other ‘-isms’ that affect people, like sexism.

Theme 1. Communities with less power don't have a say in big decisions, which means they don't get the resources they need.

1A. They don't have power in politics and decision-making.

1B. They don't have enough access to important resources, which affects communities of color more.

Theme 2. Racism in systems gives more resources to White communities than to communities of color.

2A. People experience different access to treatment, resources, and information.

2B. The needs of White and rich communities are put first over communities of color.

Theme 3. Racism in systems causes trauma and mistrust over generations.

3A. Past and present oppression hurts many generations of communities of color.

3B. Bad experiences with health services cause mental and physical trauma and make people not trust healthcare providers.

3C. There's a lack of representation, language barriers, and not enough cultural understanding in the healthcare system.

Racism in systems, along with sexism, xenophobia, homophobia, and other issues, affects the daily lives of people of color with HIV. Our findings support the need for change to reduce health inequities caused by years of social, economic, and political oppression, made worse by policy failures related to HIV and COVID-19 in the U.S.

**Methods**

*Positionality and reflexivity*

The study team, which included the academic researchers (one belonged to South Asian immigrant community, one to the Black community), research collaborator, which included an academic researcher who identifies as multiracial and one who belongs to African immigrant community, study staff (a member belonged to African immigrant communities and one who belonged to a South Asian immigrant community), and community partners (who belonged to Black, African immigrant, Latine and LGBTQIA+ communities) met regularly during project planning, implementation, analysis, and dissemination.

*Contextualizing this analysis in a larger body of work and overall strategy for community-engagement*

This analysis focuses on qualitative data collected through a community engagement effort for a study focused on the confluence of HIV, COVID-19, and SDoH.^1^ This study leverages the National Clinical Cohort Collaborative (N3C), which is a nationally-sampled electronic health records registry, to better understand the racialized inequities that have arisen with the collision of the HIV and COVID-19 epidemics in the U.S.^2,3^. Through the present approach, we sought to meaningfully engage communities in creating and co-leading research agendas in data science, specifically as it relates to HIV-related health equity research.

We had a multifaceted approach to work with our scientific and community partners through: (1) a scientific advisory committee (SAC) which consisted of members with lived experiences and content expertise identifying as community members and/or health scientists and (2) qualitative data collection focused on the different ways structural racism and SDoH have impacted racial/ethnic health disparities in HIV and COVID-19 in collaboration with our CBO partners. Thus, in this article, we present the first of two focus areas, structural racism-related findings from our qualitative data collection and analysis efforts.

*Scientific Advisory Committee*

The SAC was co-chaired by a community leader (R.S.) and an academic scientist (R.C.P.), supported by study staff coordinating the SAC meetings and scope of work. Community members of the SAC were compensated a total $5000 for their time. The SAC convened approximately monthly starting in December 2022 to September 2024. During SAC’s hour-long meetings, example discussions included: the scientific agenda co-creation for research to understand the confluence of HIV, COVID, and SDoH; receiving feedback on various research being conducted; and reviewing focus group discussion data and other data from the study to support the interpretations and conclusions.

*Qualitative Data Collection via CBOs*

We partnered with CBOs to facilitate focus group discussions (FGDs) with lay community members affected by HIV. CBOs were sampled through convenience sampling through social and professional networks of study team members. The CBOs facilitating the FGDs advocated for and served individuals who belonged to underrepresented populations of racial/ethnicity, sexual orientation, gender and/or living with or vulnerable to HIV, with a focus on health equity advocacy, HIV prevention-focused education, and community engagement. Additional information on the participating CBOs and the community members they serve can be found in **Table 1**. To boost fidelity, we held several meetings with CBO staff focused on study-specific training and orientation material as well as one-on-one meetings for clarifying questions. Organizations were also invited to take part in a FGD centered on addressing their lived experiences with how SDoH factors and structural racism impacted the racial/ethnic disparities in HIV and COVID-19. In selecting CBOs to work with, we aimed to generate diversity of various kinds, from geography of the CBO headquarters to the type of work they conducted to which underrepresented racial communities they served. Nine organizations were approached and invited to participate in the study, and eight agreed to participate. Recruitment and facilitation of the FGDs was carried out by CBO staff, with support from an academic (S.W.) with expertise in community outreach and engagement. Additional support was provided by the study staff (B.M.). Each organization was compensated $4000 for each FGD they conducted. We recommended that each CBO recruit 10-15 potential participants per FGD, so that we would have at least 6-8 participants at each FGD. Each CBO individualized how they approached potential participants, e.g., through informational flyers and/or their own method of communication; the study team provided some example fliers or email templates.

*FGD eligibility, setting, and compensation*

Focus group discussion (FGD) eligibility was determined by the following screening questions: 1) Are you at least 18 years old?, 2) Do you identify as belonging to a community that may consider itself as having disparities in HIV, either for prevention, treatment, or both?, 3) Do you self-identify as a person of color (Latine/o or Hispanic, Black/African/African American, Asian/Asian American, Arab and other Middle Eastern American, Native American, Native Hawaiians and other Pacific Islander, or Alaska Native)? The CBOs conducted the FGDs in English on Zoom, with individual participants joining from various locations in the U.S. The only non-participants present for some of the FGDs were members of the research team who were available on Zoom, with audio and video off, to provide any extra technical support needed by the CBO. We used REDCap to collect information on consent, eligibility screening, and sociodemographic data from each participant at the beginning of the FGDs. Consent was received on the REDCap survey equivalent to an oral consent. Participants were each compensated $150 e - gift cards for participating in the FGD.

*Facilitator characteristics and CBO relationships with FGD participants*

Facilitators were internally selected within each CBO. The facilitators had varying credentials, including degrees in nursing, master’s of public health, law, or community health management, and some held leadership roles in their organizations, such as a director or manager. These facilitators had significant experience conducting qualitative data collection prior to the FGDs. Slightly more than half of the facilitators identified as women. The facilitators from the CBOs had existing relationships with the FGD participants through peer-to-peer interactions, community advisory memberships and/or regular interaction with their respective organization.

*FGD guide development*

The study team developed an initial FGD guide with input and feedback from the SAC and CBOs partners, using the Healthy People’s 2030 SDoH ^4^as a theoretical framework to orient the questions and discussion with COVID-19 and HIV-related experiences. The FGD guide was iteratively refined after each FGD was conducted, and a study team member (B.M.) met each FGD facilitator ahead of their FGDs to review it and make minor edits to flow or accessibility of language. The five sections of the discussion guide included experiences with (1) SDoH and HIV, (2) SDoH and COVID-19, (3) SDoH overlap between HIV and COVID-19, (4) research priorities, and (5) intervention priorities for SDoH work in HIV and COVID-19. While the overall focus of the FGD guide was on SDoH, occasional questions regarding the relationship between structural racism, SDoH, and HIV/COVID-19 outcomes were asked. Of note, we neither used nor provided an *a priori* structural racism framework or definition during our FGDs as we had done for SDoH, as we wanted community members to be able to provide their own interpretations of the term.

*Data Collection (cont.)*

Participant recruitment continued until thematic saturation was reached.^5^

*Analysis (cont.)*

Following our thematic analysis approach,^6^ we developed a codebook to generate analysis memos, facilitating us to identify preliminary major themes. Before the transcription of the FGDs, M.H. used the FGD guide’s domains and probes, as well as the topics highlighted in the field notes, to create an initial codebook. Once, the transcripts were uploaded in Dedoose, each coder coded the same single transcript independently elaborating on the initial coding tree and came together to discuss their process and any discrepancies in the codes or interpretations of content. Thereafter, they coded the remainder of the transcripts individually, with regular meetings to check in about the coding process, incorporate any new codes, etc. One coder (B.M.) reviewed all coded transcripts to ensure consistency among them.

**Results**

*Subtheme 1A. There is a lack of power in political and decision-making spaces.* Many participants observed that those in power were also associated with wealth, which directly affects which groups are included in decision-making spaces. There was an overwhelming sentiment amongst the participants that the ones making decisions about their communities do not represent them. Participants expressed that most of the decisions made did not support their needs, and moreso, made their lives harder by, for example, having to go farther for basic resources, good healthcare, education, work, etc. As reflected in the participants’ examples, policy today is still rooted in structural racism, e.g. segregation, gerrymandering. Many participants highlighted how COVID-19 also emphasized some of these inequities that have long existed for HIV, but just made more apparent and diffuse by COVID-19.

For example, one participant expressed how many Black people did not have the same liberties as White people when it came to the pandemic because they had families to support and essentially had no other option than to continue working. This lack of choice for Black and Latine communities felt intentional because of decisions that are made at larger federal and state levels that have downstream effects disproportionately affecting these communities.

*Subtheme 1B. Insufficient and inadequate built environment infrastructure disproportionately impacting communities of color.* Overwhelmingly, participants' experiences reflected the inadequacy of their built environment that a lack of resources in almost every capacity had fostered a need for “survival living.” Participants said they were aware that the neighborhoods they live in, and the structures that limit and surround them, were intentionally made to do so decades ago. These same historical structures aligned with systems today that cause experiences such as gentrification and pushing people out, creating more crowded communities, and leading to higher chances of spread of COVID-19. One participant even details how they, and most of their community, had to travel outside of their neighborhoods for work but they have never observed white people come to work in their neighborhoods. It was clear amongst the participants that the “white neighborhoods” are associated with better access to resources, specifically a higher chance of receiving better healthcare.

*Subtheme 2A. Experiences of differential access to treatment, resources, and information.* Many of the participants' articulated their lived experience regarding differential access to resources and information specifically regarding COVID-19. The shared narratives suggest significant challenges in access to many resources (e.g., medication, education, etc.). Some participants, in the context of immigration, expressed that healthcare in the U.S. is uniquely stressful. Some mentioned that healthcare was better in other countries, and those that had first-hand experiences elsewhere noted they did not feel nearly as stressed as they do in the U.S., both about the actual care they receive and how to finance it. Discussions underscored how much of the differential access is intentionally so, and how “the system” inherently does not benefit some communities. Additionally, many emphasized the lack of comprehensive research and data, emphasizing the absence of nuanced data that reflected the experiences of their specific communities – particularly for those navigating substance use challenges and/or experiencing houselessness. This is in tandem with the fact that Black and brown individuals also know that those communities are getting more trustworthy information.

*Subtheme 2B. The needs and wellbeing of White people are prioritized above those of communities of color.* Participants discussed experiences of not being prioritized in many of the systems they interacted with, naming white supremacy and the prioritization of the wellness of white individuals and communities as an underpinning of those systems. In healthcare, this manifests in several ways, such as the inequitable distribution of resources and vaccines. For example, the feeling that Black and other communities of color were an afterthought when it came to COVID-19 vaccine distribution, was frequently discussed. Beyond healthcare, systemic inequities extend to economic mobility and access to essential and quality resources like education and transportation. People described the challenge of accessing transportation due to the cost of gas, time needed for public transportation to get to health clinics that had the resources they needed. As noted by participants, the historical patterns of exclusion, from quality education, economic opportunities, and safe, accessible transportation reinforce cycles of disadvantage, and established strategies and tactics of deprioritizing their lives are observed over and over again. As a result, communities of color face significant barriers to obtaining the information and resources necessary for well-being, perpetuating a cycle of inequality where the needs of white people are systemically prioritized, and the challenges faced by Black and brown individuals are systematically overlooked.

*Subtheme 3A. Historical and ongoing oppression negatively impacts multiple generations of communities of color.* Many participants brought up the intergenerational impact of historical structures and systems that perpetuate inequality and harm. People described generational fears and “curses”, where the trauma of past and ongoing discrimination continues to shape current experiences, such as knowledge about available resources and structures, access to education, health and wealth. Many individuals also described how current cultural norms and the lingering stigma, such as that associated with HIV, also play a significant role in the everyday decisions their communities make about their health.

*Subtheme 3B. The harmful experiences accessing health-related services leads to mental and physical trauma and lack of trust in providers and healthcare systems.* Although participants broadly discussed the negative impact of various social and economic structures in society, a key thread throughout the discussions was the harmful impact of the healthcare system in causing mental and physical trauma and distrust. Many individuals shared stories of delaying seeking treatment due to negative experiences and subsequent fear and mistrust of the providers and healthcare system. Others described feeling dismissed when they did engage in care, and not wanting to return. Many people made the connection between the historical injustices within the healthcare system and distrust, such as the neglect and stigma associated with HIV in the 1980s and 1990s, which continues to affect present-day interactions with healthcare, including their experiences with COVID-19. Additionally, many shared that they felt like the inconsistent guidelines, communication, and health directives contributed to both mental and physical health stressors and making it harder to trust health authorities, further exacerbating the problem.

*Subtheme 3C. Lack of representation, language barriers, accessible services, and cultural humility, sensitivity, and competency at multiple levels of the healthcare system.* A significant number of participants agreed that their interactions with various healthcare systems lacked cultural competency, sensitivity, and humility. According to many participants, there is a clear need for healthcare workers to receive more training on how to engage respectfully with communities outside their own.

Communication and language barriers also remain significant, as many healthcare facilities still lack the necessary infrastructure to provide appropriate care. This stems from the broader lack of community representation in the healthcare workforce, which hinders providers' ability to engage with patients in a culturally competent manner and, in turn, makes it more difficult for patients to feel safe and welcomed. This, coupled with the underrepresentation of these communities in the healthcare workforce, limits providers' ability to interact in a culturally competent manner and makes it harder for patients to feel safe and welcomed.

**References**

Seyedroudbari S, Ghadimi F, Grady G, et al. Assessing Structural Racism and Discrimination Along the Pre-exposure Prophylaxis Continuum: A Systematic Review. *AIDS Behav*. 2024;28(9):3001-3037. doi:10.1007/s10461-024-04387-y

National Clinical Cohort Collaborative (N3C) Homepage. <https://n3c.cd2h.org/>

Bennett TD, Moffitt RA, Hajagos JG, et al. The National COVID Cohort Collaborative: Clinical Characterization and Early Severity Prediction. *medRxiv*. Published online January 23, 2021:2021.01.12.21249511. doi:10.1101/2021.01.12.21249511

Social Determinants of Health - Healthy People 2030 | health.gov. Accessed July 19, 2024. <https://health.gov/healthypeople/priority-areas/social-determinants-health>

Ahmed SK. Sample size for saturation in qualitative research: Debates, definitions, and strategies. *J Med Surg Public Health*. 2025;5:100171. doi:10.1016/j.glmedi.2024.100171

Braun V, Clarke V. Using thematic analysis in psychology. *Qual Res Psychol*. 2006;3(2):77-101. doi:10.1191/1478088706qp063oa
